# Selective Pressure of Antibiotic Pollution on Bacteria of Importance to Public Health

**DOI:** 10.1289/ehp.1104650

**Published:** 2012-05-08

**Authors:** Alfredo Tello, Brian Austin, Trevor C Telfer

**Affiliations:** Institute of Aquaculture, University of Stirling, Stirling, Scotland, United Kingdom

**Keywords:** antibiotic pollution, antibiotic resistance, minimum inhibitory concentration distributions, risk assessment, species sensitivity distributions

## Abstract

Background: Many bacteria of clinical importance survive and may grow in different environments. Antibiotic pollution may exert on them a selective pressure leading to an increase in the prevalence of resistance.

Objectives: In this study we sought to determine whether environmental concentrations of antibiotics and concentrations representing action limits used in environmental risk assessment may exert a selective pressure on clinically relevant bacteria in the environment.

Methods: We used bacterial inhibition as an assessment end point to link antibiotic selective pressures to the prevalence of resistance in bacterial populations. Species sensitivity distributions were derived for three antibiotics by fitting log-logistic models to end points calculated from minimum inhibitory concentration (MIC) distributions based on worldwide data collated by the European Committee on Antimicrobial Susceptibility Testing (EUCAST). To place bacteria represented in these distributions in a broader context, we performed a brief phylogenetic analysis. The potentially affected fraction of bacterial genera at measured environmental concentrations of antibiotics and environmental risk assessment action limits was used as a proxy for antibiotic selective pressure. Measured environmental concentrations and environmental risk assessment action limits were also directly compared to wild-type cut-off values.

Results: The potentially affected fraction of bacterial genera estimated based on antibiotic concentrations measured in water environments is ≤ 7%. We estimated that measured environmental concentrations in river sediments, swine feces lagoons, liquid manure, and farmed soil inhibit wild-type populations in up to 60%, 92%, 100%, and 30% of bacterial genera, respectively. At concentrations used as action limits in environmental risk assessment, erythromycin and ciprofloxacin were estimated to inhibit wild-type populations in up to 25% and 76% of bacterial genera.

Conclusions: Measured environmental concentrations of antibiotics, as well as concentrations representing environmental risk assessment action limits, are high enough to exert a selective pressure on clinically relevant bacteria that may lead to an increase in the prevalence of resistance.

Antibiotic pollution may facilitate the development and spread of antibiotic resistance (Martinez 2008). Antibiotics used in human and veterinary medicine can enter the environment via wastewater treatment plant effluents, hospital and processing plant effluents, application of agricultural waste and biosolids to fields, and leakage from waste-storage containers and landfills (Kümmerer 2009a; Sarmah et al. 2006). One of the difficulties of relating increased levels of resistance in the environment to antibiotic pollution, however, is the fact that antibiotic resistance genes can be co-released into the environment with antibiotic compounds (Kümmerer 2009b). The question then is whether an observed increase in resistance emerged as a result of the selective pressure of the antibiotic in the environment or if it emerged within the treated host.

Antibiotic resistance genes and antibiotic compounds are different pollutants that have different modes of action and are subject to different fate processes in the environment (Martinez 2009). They are also likely to respond differently to treatment processes designed to remove them from environmental compartments and from liquid and solid wastes (Pei et al. 2007). Estimating the relative contribution of pollution by antibiotic resistance genes and antibiotic compounds to increased levels of antibiotic resistance is important, as this knowledge may be used to improve the effectiveness of counteractive measures.

Except for the the report by Kristiansson et al. (2011), who found excessive concentrations of antibiotics and concurrent high levels of resistance in streams receiving effluents from a drug-production plant in India, there is limited evidence as to whether environmental concentrations of antibiotics can enhance the development and spread of resistance in the environment (e.g., Knapp et al. 2008). Current guidelines on the environmental risk assessment of medicinal products for human and veterinary use in the European Union, for example, do not explicitly address the effect of antibiotics on the prevalence of antibiotic resistance in the environment [European Medicines Agency (EMEA) 2006, 2008].

From an environmental health perspective, the selective pressure that antibiotic pollution may exert on clinically important bacteria is of particular concern. Several clinically relevant bacteria, such as *Escherichia coli* and the enterococci, occur and are able to grow in different environments (Moriarty et al. 2008; Topp et al. 2003). In the presence of environmental concentrations of antibiotics, they may face a selective pressure leading to a gradual increase in the prevalence of resistance.

In the present study we used bacterial species sensitivity distributions derived from a comprehensive set of minimum inhibitory concentration (MIC) distributions of antibiotics to model bacterial sensitivities and characterize the selective pressure that antibiotic pollution may exert on bacteria of importance to public health that are found in the environment. The study was carried out under the premise that antibiotics will primarily increase the prevalence of resistance by favoring the selection of resistant phenotypes via the inhibition of sensitive ones. Although there is evidence to suggest that subinhibitory concentrations of antibiotics may indirectly favor resistance (Hoffman et al. 2005), the use of bacterial inhibition as an assessment end point provides a standardized response across taxa that can be directly linked to a selective pressure favoring an increase in the prevalence of resistance.

We derived species sensitivity distributions for three antibiotics from publicly available MIC distributions and determined the fraction of inhibited bacterial taxa at antibiotic concentrations that were measured in different environments and used as action limits in environmental risk assessment. To our knowledge, this is the first study in which measured environmental concentrations of antibiotics are examined in regard to the antibiotic sensitivity of clinically relevant bacteria.

## Methods

*MIC distributions.* We obtained MIC distributions for ciprofloxacin, erythromycin, and tetracycline from the European Committee on Antimicrobial Susceptibility Testing (EUCAST) MIC and zone diameter distribution website (EUCAST 2010; Kahlmeter et al. 2003). Distributions are based on data collated from > 20,000 different worldwide sources and encompass the variability within species and between researchers, methods, and geographic areas.

Ciprofloxacin, erythromycin, and tetracycline were selected from among a list of approximately 150 compounds in the database for three reasons: *a*) they represent three distinct classes of antibiotics of importance to human and veterinary medicine; *b*) the number of bacterial taxa represented in their MIC distributions was higher than in most of the other compounds in the database; and *c*) they have been measured in different environmental compartments.

*Phylogenetic analysis.* To place bacteria represented in the EUCAST MIC distributions in a wider phylogenetic context, we conducted a brief phylogenetic analysis. 16S rRNA sequences from bacterial taxa represented in the MIC distributions were obtained from the All-Species Living Tree Project (LTP; Yarza et al. 2008), March 2011 release, and imported into ARB software (Ludwig et al. 2004). We selected bacterial taxa represented in the MIC distributions of ciprofloxacin, erythromycin, and tetracycline to create a pooled 16S rRNA sequence alignment comprising the species represented in the MIC distributions of all three antibiotics. Some bacterial taxa were not represented in the LTP database; therefore, the alignment contained a subset of the taxa represented in each original MIC distribution [see Supplemental Material, Table S1 (http://dx.doi.org/10.1289/ehp.1104650)]. This alignment, along with the entire LTP alignment for the domain *Bacteria*, was imported into the ape package (version 2.6-2) of the R environment for statistical computing (Paradis and Strimmer 2004); we then calculated an evolutionary distance matrix for each alignment using Kimura’s two-parameter substitution model (Kimura 1980). An unrooted phylogenetic dendrogram was estimated from the evolutionary distance matrix of the pooled antibiotic alignment using the neighbor-joining method (Saitou and Nei 1987), and confidence was assessed by bootstrapping with 1,000 permutations. We plotted histograms of pairwise evolutionary distances covered by the pooled 16S rRNA alignment of species represented in the MIC distributions of all three antibiotics and by the entire LTP alignment for the domain *Bacteria* to assess the range of evolutionary distances covered by our data set in relation to that of all sequenced type strains of bacteria.

*Bacterial species sensitivity distributions.* End point selection. Species sensitivity distributions were derived using the median MIC (MIC_50_) and the no observed effect MIC (NOEC) of each taxon. MIC tests are performed using double-dilution steps of antibiotic concentrations, and the data they generate is interval censored. Therefore, we considered the conservative MIC_50_ of each species to be the antibiotic concentration immediately below the observed 50th percentile, and the NOEC to be the antibiotic concentration immediately below the lowest MIC observed in each taxon. MIC_50_ and NOEC values were aggregated within cogeneric species by using the arithmetic mean to minimize the lack of independence between individual observations [see Supplemental Material, Section 1 and Figure S1 (http://dx.doi.org/10.1289/ehp.1104650)]. To derive species sensitivity distributions, we used pooled MIC_50_ and NOEC values only from genera for which there was evidence to suggest that—under certain conditions—the genera could grow in an environmental compartment (e.g., soil, sewage, fresh water) (for details regarding the selection of genera, see Supplemental Material, Section 2 and Table S2).

**Figure 1:**
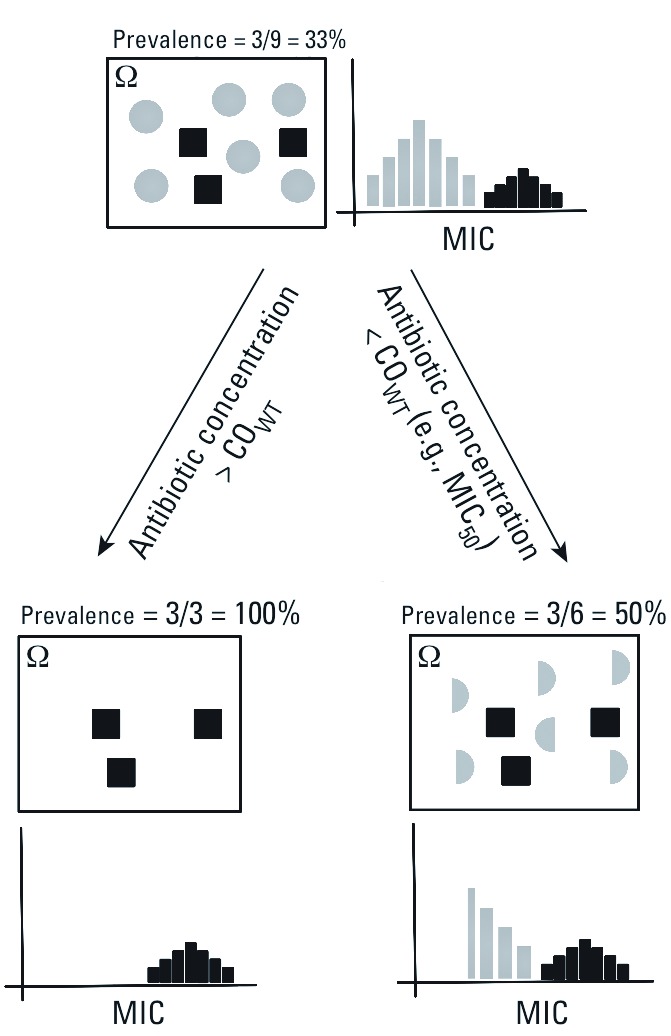
Conceptual link between the MIC_50_ and antibiotic concentrations > CO_WT_ with resistance prevalence in a universe (Ω) including resistant (black) and wild-type (gray) populations, and its relation to the MIC distribution. Antibiotic concentrations > CO_WT_ completely inhibit wild-types, and resistance prevalence in the active (i.e., growing) population will be 100%. Concentrations of antibiotics ≤ CO_WT_, such as the MIC_50_, will inhibit a fraction of the wild-type population (e.g., 50%). If we assume equal growth rates of wild-type and resistant populations, the prevalence of antibiotic resistance will increase in the active population.

Linking end points to resistance. The MIC_50_ was calculated including data beyond the wild-type cut-off value (CO_WT_). The CO_WT_ separates microorganisms with (i.e., non–wild-type) and without (i.e., wild-type) acquired resistance mechanisms and represents an antibiotic concentration above which only bacteria with acquired resistance mechanisms can grow (Kahlmeter et al. 2003) A comparison of MIC_50_ values with the wild-type MIC range of species in the EUCAST distributions indicates that the MIC_50_ is an adequate estimate of the wild-type MIC (i.e., it falls within the wild-type MIC range in those species that have one), except for a few cases in which it falls above the wild-type MIC range. Concentrations of antibiotics ≥ MIC_50_ are therefore likely to inhibit approximately half of the wild-type population. Assuming equal growth rates of wild-type and resistant populations, this causes an increase in the prevalence of resistance in the remaining active populations (Figure 1). In contrast, the NOEC represents a minority of isolates across taxa whose MIC values are sometimes below the MIC range representative of the wild-type population; we used the NOEC as a means to assess the lower limit of antibiotic sensitivity represented in the MIC distributions.

Bootstrap regression. We derived species sensitivity distributions by bootstrap regression, as described by Grist et al. (2002). The MIC_50_ and NOEC vectors of each antibiotic were resampled 5,000 times. To each of these bootstrap resamples, a log-logistic model was fitted by maximum likelihood estimation of the distribution parameters and direct optimization of the log-likelihood function following the method of Nelder and Mead (1965) and using the fitdistrplus package, version 0.1–4, of the R environment for statistical computing (Delignette-Muller et al. 2010). The distribution parameters α (i.e., location) and β (i.e., scale) from each fitted curve were used to derive 5,000 replicate estimates of antibiotic concentrations associated with a potentially affected fraction between percentiles 0.01 and 0.99 at 0.01-step intervals. From these, the bootstrap estimate and 95% bootstrap confidence intervals (CIs) were calculated. The MIC distributions used in this study, along with an R script to replicate the analysis, are available from the authors.

We determined the potentially affected fraction of bacterial genera by all three antibiotics at the aquatic and soil VICH phase I action limits [International Cooperation on Harmonisation of Technical Requirements for Registration of Veterinary Medicinal Products (VICH) 2000] and at measured environmental concentrations reported in the literature (e.g., Kolpin et al. 2002). This was complemented with a direct comparison of measured environmental concentrations and VICH action limits with the CO_WT_ of species represented in the MIC distributions of each antibiotic. Although ciprofloxacin is not approved for use in veterinary medicine, it is the major active metabolite of enrofloxacin in different species (Idowu et al. 2010). In the absence of data for enrofloxacin, we used ciprofloxacin as a representative of the fluoroquinolones in comparisons of potentially affected fractions and VICH phase I action limits. We obtained measured environmental concentrations of ciprofloxacin, erythromycin, and tetracycline from Kolpin et al. (2002), Golet et al. (2002), and Luo et al. (2011), and from data collated by Hamscher (2006). Antibiotic concentrations are expressed as parts per billion to facilitate analysis and comparisons.

## Results

**Figure 2:**
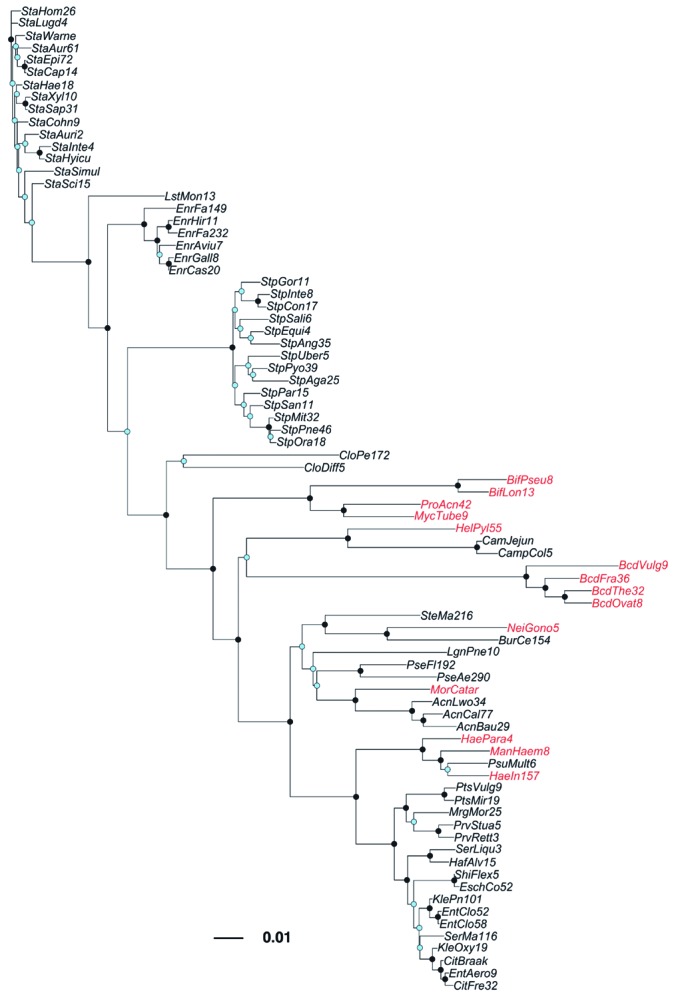
Unrooted neighbor-joining dendrogram of species (by LTP name) represented in the pooled 16S rRNA alignment of ciprofloxacin, erythromycin, and tetracycline. Species highlighted in red were not included in the species sensitivity distributions due to lack of evidence of growth in the environment. Bar units indicate the number of nucleotide substitutions per site. Black nodes indicate ≥ 70% bootstrap support; blue nodes indicate < 70% bootstrap support. Full species names are available in Supplemental Material, Table S1 (http://dx.doi.org/10.1289/ehp.1104650).

**Figure 3:**
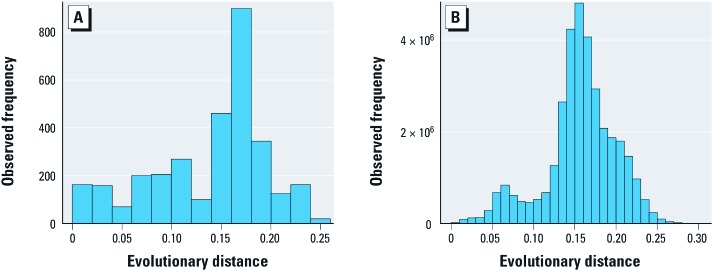
Histograms showing the range of pairwise evolutionary distances covered by (*A*) the pooled 16S rRNA alignment of species represented in the MIC distributions of all three antibiotics, and (*B*) the entire LTP 16S rRNA alignment for the domain *Bacteria*. Pairwise evolutionary distances in *A*were calculated from the same 16S rRNA alignment used to construct the dendrogram in Figure 2.

*Phylogenetic and environmental overview of MIC distributions.* Seventy-nine species from the ciprofloxacin, erythromycin, and tetracycline MIC distributions were represented in the LTP 16S rRNA database [Figure 2; see also Supplemental Material, Table S1 (http://dx.doi.org/10.1289/ehp.1104650)]. Major bacterial groups in Figure 2 (appearing in order from top to bottom in the dendrogram) include staphylococci, enterococci, streptococci, a few representatives of the Actinobacteria (e.g., *Clostridium* spp., *Mycobacterium* spp.), *Bacteroides*, pseudomonads (e.g., *Pseudomonas* spp., *Burkholderia* spp.), and the enterics (e.g., *Escherichia* spp., *Enterobacter* spp., *Proteus* spp., *Klebsiella* spp.). The range of evolutionary distances covered by these species spans the range of evolutionary distances represented in the entire LTP database, highlighting it as a rather diverse phylogenetic group (Figure 3).

Bacterial genera used to derive the species sensitivity distribution of each antibiotic represent commensal and pathogenic bacteria that occur, and may grow, to a larger or lesser extent in the environment (Table 1). Among the 27 genera included in the species sensitivity distributions are some known to be widely distributed in the environment, such as *Pseudomonas*, *Acinetobacter, Burkholderia*, and *Chryseobacterium* (Madigan et al. 2009; Vandamme et al. 1994), as well as others for which growth in the environment has either been reported or for which there is evidence to suggest that under certain conditions it is likely to occur [see Supplemental Material, Table S2 (http://dx.doi.org/10.1289/ehp.1104650)].

**Table 1:** Bacterial genera included in the species sensitivity distribution of each antibiotic.

Genus	Cipro	Eryth	Tetra
1	Acinetobacter		+		–		+
2	Alcaligenes		+		–		–
3	Burkholderia		+		–		–
4	Campylobacter		+		+		+
5	Chryseobacterium		+		–		–
6	Citrobacter		+		–		+
7	Clostridium		–		+		+
8	Enterobacter		+		–		+
9	Enterococcus		+		+		+
10	Escherichia		+		–		+
11	Hafnia		+		–		+
12	Klebsiella		+		–		+
13	Kluyvera		+		–		+
14	Legionella		+		+		–
15	Listeria		+		–		+
16	Morganella		+		–		+
17	Pasteurella		+		+		+
18	Proteus		+		–		+
19	Providencia		+		–		–
20	Pseudomonas		+		–		+
21	Raoultella		+		–		+
22	Salmonella		+		–		+
23	Serratia		+		–		+
24	Staphylococcus		+		+		+
25	Stenotrophomonas		+		–		+
26	Streptococcus		+		+		+
27	Yersinia		+		–		+
Abbreviations: Cipro, ciprofloxacin; Eryth, erythromycin; Tetra, tetracycline. “+” and “–” symbols indicate presence or absence, respectively, of the genera in each data set.							

**Figure 4:**
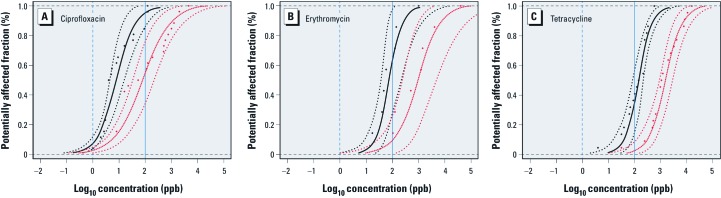
Species sensitivity distributions derived for the NOEC (black) and MIC_50_ (red) curves with overlayed empirical cumulative distributions (dots). Fitted curves represent the bootstrap estimate and 95% bootstrap CIs for the log-logistic model. Dashed and solid blue vertical lines represent the VICH phase I aquatic and soil action limits, respectively. (*A*) Ciprofloxacin: NOEC (*R*^2^ = 0.90; *p* < 0.0001), MIC_50_ (*R*^2^ = 0.91; *p < *0.0001); bootstrap estimate of model parameters: NOEC species sensitivity distribution (α = –2.1, β = 0.37), MIC_50_ species sensitivity distribution (α = –1.0, β = 0.54). (*B*) Erythromycin: NOEC (*R*^2^ = 0.96; *p <*0.001), MIC_50_ (*R*^2^ = 0.97; *p < *0.0001); bootstrap estimate of model parameters: NOEC species sensitivity distribution (α = –1.13, β = 0.29), MIC_50_ species sensitivity distribution (α = –0.014, β = 0.48). (*C*) Tetracycline: NOEC (*R*^2^ = 0.97; *p <*0.0001), MIC_50_ (*R*^2^ = 0.98; *p < *0.0001); bootstrap estimate of model parameters: NOEC species sensitivity distribution (α = –0.84, β = 0.27), MIC_50_ species sensitivity distribution (α = 0.2, β = 0.33). The potentially affected fraction of bacterial genera at a given antibiotic concentration is read from the *y*-axis at the point in which the antibiotic concentration intersects with the species sensitivity distribution. For example, a concentration of 100 ppb (i.e., log_10_ concentration = 2) of ciprofloxacin inhibits approximately one-half of the wild-type population (i.e., red MIC_50_ curve) in 54% of the bacterial genera and at least some individuals (i.e., black NOEC curve) in 95% of the bacterial genera.

*Inhibitory effects at environmental concentrations.* The log-logistic model had a good fit to the NOEC and MIC_50_ vectors, explaining ≥ 90% of the variance in the original data (Figure 4). Table 2 shows that the potentially affected fraction of bacterial genera at measured environmental concentrations of ciprofloxacin, erythromycin, and tetracycline in water environments—including surface water, sewage treatment plant (STP) effluents, raw sewage, and groundwater—is low, with upper 95% CIs of 7% and 3.2% in raw sewage for ciprofloxacin using the NOEC and MIC_50_ species sensitivity distributions, respectively. At the low range of concentrations measured in surface waters and STP effluents, the practical difference between potentially affected fraction estimates is minimal using the NOEC and MIC_50_ species sensitivity distributions. For erythromycin, the NOEC and MIC_50_ species sensitivity distributions overlap at the lower tail of the distributions, causing the MIC_50_ species sensitivity distribution to estimate slightly higher potentially affected fractions than the NOEC species sensitivity distribution for concentrations measured in surface waters and STP effluents. Given that the potentially affected fractions of both species sensitivity distributions are well below 1% at this range of concentrations, this discrepancy was not significant for our assessment of effects.

**Table 2:** Potentially affected fraction for each antibiotic at measured environmental concentrations and environmental risk assessment action limits using the NOEC and MIC50 species sensitivity distributions.

Potentially affected fraction (95% CI)		
Antibiotic/environment		Concentration (ppb)	NOEC species sensitivity distribution	MIC_50_ species sensitivity distribution
Ciprofloxacin							
	Surface water		0.03a		0.1 (0.008, 0.4)		0.2 (0.03, 0.4)
			0.36b		2.3 (0.7, 4.3)		1.2 (0.4, 2.3)
	River sediments		48b		89 (75, 99)		40 (23, 60)
	STP effluent		0.37b		2.4 (0.7, 4.4)		1.2 (0.4, 2.3)
			0.062c		0.3 (0.03, 0.8)		0.3 (0.1, 0.7)
			0.11c		0.6 (0.08, 1.3)		0.4 (0.1, 1)
	Raw sewage		0.313c		2 (0.5, 3.8)		1.1 (0.4, 2)
			0.568c		4 (1.6, 7)		1.7 (0.6, 3.2)
	Swine feces lagoon sediment		340b		99 (95, 100)		76 (59, 92)
	VICH phase I limit (aquatic)		1		8 (4, 12)		2.7 (1, 4.7)
	VICH phase I limit (terrestrial)		100		95 (86, 100)		54 (36, 76)
Erythromycin							
	Surface water		0.024b		0.0001 (0, 0.02)		0.003 (0, 0.09)
	River sediments		19b		8.6 (0.5, 23)		2 (0.03, 7.6)
	STP effluent		0.07b		0.0008 (0, 0.07)		0.008 (0, 0.2)
	Swine feces lagoon sediment		80b		53 (21, 92)		7.9 (0.4, 21)
	VICH phase I limit (aquatic)		1		0.07 (0, 1)		0.1 (0, 1)
	VICH phase I limit (terrestrial)		100		62 (27, 97)		9.7 (0.5, 25)
Tetracycline							
	Surface water		0.11a		0.0006 (0, 0.03)		0.0003 (0, 0.01)
			0.42b		0.006 (0, 0.2)		0.002 (0, 0.04)
	River sediments		73b		24 (8, 44)		1.6 (0.3, 6)
	STP effluent		0.16d		0.001 (0, 0.05)		0.0005 (0, 0.02)
			0.98d		0.02 (0, 0.4)		0.005 (0, 0.09)
			0.09b		0.0004 (0, 0.02)		0.0002 (0, 0.009)
	Swine feces lagoon sediment		1,100b		97 (92, 99)		38 (22, 56)
	Liquid manure		66,000d		100 (100, 100)		99 (97, 100)
	Farmed soil		443d		86 (77, 94)		15 (6.6, 30)
	Groundwater		0.13d		0.0008 (0, 0.04)		0.0004 (0, 0.01)
	VICH phase I limit (aquatic)		1		0.02 (0, 0.4)		0.005 (0, 0.09)
	VICH phase I limit (terrestrial)		100		35 (14, 55)		2.4 (0.5, 8.1)
aOccurrence data (maximum measured concentrations) from Kolpin et al. (2002). bOccurrence data from supporting information, Table S6, of Luo et al. (2011). Concentrations greater than the limit of detection were averaged over sampling stations for sites “Tributaries Water, Dec 2009,” “Tributaries Sediment Dec 2009,” “Pollution source water Dec 2009” for S1 and S2, and “Source Sediments Dec 2009” for S3 and S4; surface water concentrations and river sediment concentrations are from corresponding sampling sites. cOccurrence data (mean measured concentrations) from Golet et al. (2002). dOccurrence data (maximum measured concentrations) collated by Hamscher (2006).							

Potentially affected fractions in river sediments, swine feces lagoons, liquid manure, and farmed soil are markedly higher than those in aquatic compartments (Table 2). Concentrations of ciprofloxacin, erythromycin, and tetracycline measured in river sediments are ≥ MIC_50_ values of ≤ 60%, 7.6%, and 6% of the bacterial genera, respectively (i.e., upper 95% CIs in Table 2). Estimated concentrations of these three antibiotics in the sediments of a swine feces lagoon are ≥ MIC_50_ values of ≤ 92%, 21%, and 56% of the bacterial genera. The extremely high concentration of tetracycline in liquid manure reported by Hamscher (2006) is ≥ MIC_50_ of 100% of the bacterial genera. In contrast, the high concentration of tetracycline measured in farmed soil is ≥ MIC_50_ for ≤ 30% of genera. We estimated that the tetracycline concentration reported by Hamscher (2006) for farmed soil inhibits at least some isolates in up to 94% of the bacterial genera (i.e., NOEC species sensitivity distribution). Some environments, such as soil and sediments, are likely to contain more bacterial genera of clinical relevance than others. Thus, for example, a potentially affected fraction of 30% for tetracycline in farmed soil may inhibit more bacterial genera than a potentially affected fraction of 100% in liquid manure and therefore have larger public health implications.

**Figure 5:**
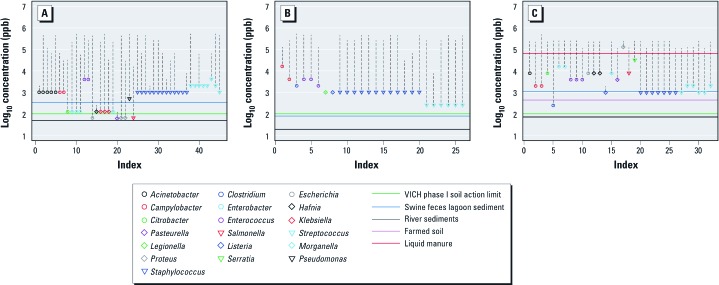
MICs ≥ CO_WT_ for bacterial taxa in the MIC distributions of ciprofloxacin (*A*), erythromycin (*B*), and tetracycline (*C*). Symbols represent the CO_WT_ for different genera; dashed vertical lines extend up to the maximum MIC beyond the CO_WT_; and horizontal lines represent antibiotic concentrations. The *x*-axis represents the number of bacterial taxa with a defined CO_WT_ for each antibiotic, and the *y*-axis indicates antibiotic concentrations.

For bacterial taxa represented in the MIC distribution of each antibiotic, we compared MIC values ≥ CO_WT_ with measured environmental concentrations (Figure 5).Concentrations > CO_WT_ for a given bacterial taxa completely inhibit the wild-type population, increasing the prevalence of resistance in the remaining active population to 100%. The measured environmental concentration of ciprofloxacin in swine feces lagoon sediment is > CO_WT_ for 14 bacterial taxa belonging to nine genera of predominantly enteric bacteria (Figure 5A). The concentration of tetracycline measured in liquid manure is > CO_WT_ for all but one bacterial taxa, and the concentration measured in swine feces lagoon sediment is borderline with the CO_WT_ for *Staphylococcus* and *Streptococcus* (Figure 5C). Measured environmental concentrations of erythromycin are < CO_WT_ for all taxa (Figure 5B).

*Inhibitory effects at VICH phase I action limits.* Potentially affected fractions at the VICH phase I aquatic action limit suggest that the action limit is protective of major inhibitory effects on bacteria by all three antibiotics (Figure 4), although a minority of sensitive individuals could be inhibited in up to 12% of genera (i.e., upper 95% CI in ciprofloxacin NOEC species sensitivity distribution) (Table 2). The 1-ppb VICH phase I aquatic action limit refers to an environmental introduction concentration (i.e., concentration in an effluent); thus, exposure concentrations in receiving water bodies are further reduced by dilution.

Concentrations of ciprofloxacin and erythromycin at the VICH phase I soil action limit are estimated to be ≥ MIC_50_ values of ≤ 76% and 25% of bacterial genera, respectively (Figure 4, Table 2). Figure 4 shows that the VICH phase I soil action limit is below the erythromycin and tetracycline CO_WT_ for all species, indicating that at this concentration these antibiotics are not expected to inhibit 100% of the wild-type population in any species. Conversely, the ciprofloxacin MIC distributions (Figure 5A) show that the 100-ppb soil action limit is > CO_WT_ for five bacterial taxa and borderline with that for nine other taxa.

It is also illustrative to consider these action limits in relation to the empirical MIC_50_ of individual species. The MIC_50_ for ciprofloxacin, for example, is 8 ppb in *E. coli* (*n* = 17,877), 8 ppb in *Enterobacter cloacae* (*n* = 2,354), 64 ppb in *Acinetobacter lwoffi* (*n* = 262), 125 ppb in *Pseudomonas aeruginosa* (*n* = 27,387), and 125 ppb in *Campylobacter coli* (*n* = 2,532). The MIC_50_ for erythromycin is 32 ppb in *Streptococcus pneumoniae* (*n* = 40,452) and 125 ppb in *Staphylococcus aureus* (*n* = 36,038). The MIC_50_ for tetracycline is 32 ppb in *Clostridium difficile* (*n* = 832) and 125 ppb in *Str. pneumoniae* (*n* = 13,813). These MIC_50_ are between 12.5 times lower to slightly higher than the 100-ppb soil action limit. Several species also have NOECs that are orders of magnitude lower than the 100-ppb soil threshold.

## Discussion

In this study we found that environmental concentrations of antibiotics, as well as concentrations representing action limits used in the environmental risk assessment of veterinary medicines, may be high enough to inhibit growth in bacteria of clinical importance occuring in different environments. By completely or partially inhibiting the growth of wild-type bacterial populations, antibiotics cause a selective pressure that will increase the prevalence of resistance. The potentially affected fractions for ciprofloxacin, erythromycin, and tetracycline at measured environmental concentrations of river sediments, swine feces lagoon sediments, liquid manure, and farmed soil suggest that these environments are likely to be hot spots for the selection of resistance. In this regard, the comparison of measured environmental concentrations of ciprofloxacin and tetracycline and their respective CO_WT_ values in different bacteria is striking (Figure 5) because it shows that wild-type populations of certain species are completely inhibited at these concentrations *in vitro*. In swine feces lagoons, liquid manure, and soil amended with manure, concentrations of certain antibiotics can build up to levels that may act to extend the antibiotic selective pressure that started for some bacteria within their treated hosts and exert a new selective pressure on other bacteria. Interestingly, in a study of soil from the 1970s to the present, Knapp et al. (2010) found a significant increase in tetracycline resistance genes that mimicked the use of tetracyclines in agriculture in the Netherlands. Although studies have generally failed to find a significant effect of tetracyclines on resistance levels in soil (e.g., Agersø et al. 2006), some evidence suggests that it could have contributed to the persistence and prevalence of resistance genes (Schmitt et al. 2006). Our results indicate that tetracycline concentrations in soil may build up to levels high enough to exert a significant selective pressure on clinically relevant bacteria.

The extrapolation of MIC data to the field has inherent limitations that must be considered in the interpretation of our results. Physicochemical and biological conditions of MIC tests, for example, are not representative of those generally encountered by bacteria in the environment. MIC tests are also acute tests, whereas the exposure to antibiotics in the environment is mainly chronic and will exert a constant selective pressure over extended periods of time (Kümmerer 2009a; Sarmah et al. 2006). Chronic exposure provides a longer temporal window for the selective enrichment of resistance and will favor stepwise transitions from low-level to high-level clinical resistance. In this study we did not formally address the bioavailability of antibiotics in the environment. However, tetracyclines and macrolides have been shown to retain their bioactivity and inhibit bacterial growth even when tightly adsorbed by clay particles (Chander et al. 2005); there is also evidence to suggest that fluoroquinolones retain part of their activity when sorbed to solids (Córdova-Kreylos and Scow 2007). In the environment, bacteria are likely exposed to multiple antibiotics and other substances, such as metals and disinfectants, which will affect the selection of resistance. Synergistic and/or antagonistic interactions between combinations of antibiotics, for example, may significantly influence the evolution of resistance (Michel et al. 2008). Because many antibiotic resistance genes are associated with mobile genetic elements carrying multiple antibiotic resistance genes and genes conferring resistance to heavy metals and/or disinfectants (Chopra and Roberts 2001; Ciric et al. 2011), any of these factors may select for multidrug resistance. An example relevant to this study is the broad host range transposon Tn1545, which encodes resistance to tetracycline, erythromycin, and kanamycin (Clewell et al. 1995). In all, these factors emphasize the complexity of relating antibiotic pollution to the prevalence of antibiotic resistance in the environment, and inevitably introduce a degree of uncertainty in our results. Despite these limitations, however, our results provide a means to grasp the potential effect of antibiotic pollution on the prevalence of resistance in clinically relevant bacteria in the environment by putting measured environmental concentrations in perspective with bacterial sensitivities.

The link between MIC_50_ and resistance is based on the assumption that antibiotic concentrations ≤ CO_WT_ may increase the prevalence of resistance to < 100% by inhibiting a fraction of the wild-type population. Variation within the wild-type part of MIC distributions is normally in the order of 3–5 log_2_ MIC steps (Schön et al. 2009). Although this variation may reflect inherent variation in antibiotic sensitivity and other biological features that influence the MIC, it may also reflect method variability. In environmental compartments such as those discussed in this study, it is reasonable to expect some degree of inherent variation in the antibiotic sensitivity of wild-type populations. Environments such as sewage, river sediments, and agricultural soil act as transient or permanent sinks in which wild-type populations of the same species, from different sources, and with slightly differing sensitivities may physically converge. These environments are also likely to have microgradients of physicochemical variables, such as pH and nutrients, that are known to affect the MIC of bacteria *in vitro* (Bonfiglio and Livermore 1991; Butler et al. 2001). Collectively, these factors may provide enough variation in antibiotic sensitivity to enable the differential inhibition of wild-type populations under equal measured environmental concentrations of antibiotics.

The VICH phase I guidance document (VICH 2000) informs environmental risk assessment of veterinary medicines and has been implemented in the regulatory scheme in the European Union, United States, Japan, and Australia (de Knecht et al. 2009). Under VICH phase I guidance, the environmental risk assessment of a veterinary medicine—except for parasiticides—is discontinued if the medicine’s introduction concentration into the aquatic environment is < 1 ppb (i.e., aquatic action limit). For terrestrial environments, environmental risk assessment is discontinued if the predicted environmental concentration in soil is < 100 ppb (i.e., soil action limit). Our results suggest that the VICH phase I soil action limit for veterinary medicines does not protect background antibiotic resistance levels. Certain antibiotics at concentrations < 100 ppb may inhibit a significant fraction of clinically relevant bacteria in the environment (Figure 4); the high potentially affected fractions for erythromycin and ciprofloxacin at the 100-ppb soil threshold are a clear example of this. In a critique of action limits, Montforts (2005) used MIC data from 13 soil microorganisms and 22 antimicrobials to construct a substance–species sensitivity distribution; on the basis of this distribution, he determined that the aquatic and soil action limits should be set at 4 × 10^–4^ and 1 ppb, respectively, if they were to be protective for all compounds. Similarly, our results suggest that VICH phase I action limits leave an ample margin for antibiotics to exert a selective pressure on bacteria of clinical importance in the environment.

Current knowledge on the presence and mechanisms of bacterial resistance of clinical and environmental origin clearly indicate that the resistome of pathogens is and will continue to be inevitably linked with the environment (Martinez 2008; Wright 2010). Moreover, as this study shows, the prevalence of resistance in bacteria of importance to public health has the potential to be increased by antibiotic pollution in the environment. Therefore, to minimize the potential effect of antibiotic pollution on antibiotic resistance, resistance—or a proxy thereof—should be considered in environmental risk assessment of human and veterinary antibiotics.

MIC distributions are at the center of clinical microbiology. In conjunction with drug pharmacokinetics, MIC distributions are used to establish clinical breakpoints for the effective treatment of infectious diseases (MacGowan and Wise 2001; Turnidge and Paterson 2007). Similarly, we suggest that MIC distributions can be used to explicitly link environmental concentrations of antibiotics with the prevalence of resistance, and can therefore provide a cogent framework to address the potential effects of antibiotics on antibiotic resistance in the initial phase of a risk assessment. Just as pharmacokinetics provides information on the fate of antibiotics in the body, environmental exposure assessment can be used to further refine the assessment of effects. If necessary, MIC distributions may be used to set breakpoints to protect background resistance levels in the environment.

## Conclusions

Antibiotics are present in different environments as a result of their use in human and veterinary medicine; concentrations currently found in different environments and concentrations representing action limits used in environmental risk assessment may be high enough to exert a significant selective pressure on clinically relevant bacteria. The potentially affected fraction of bacterial genera at concentrations of antibiotics measured in river sediments, liquid manure, and farmed soil suggests that these environments are likely to be hot spots for the development of resistance. Both explicit consideration of antibiotic resistance in the environmental risk assessment of antibiotics and efforts to reduce the input of antibiotics into the environment—by limiting use and/or improving the treatment of liquid and solid wastes—are crucial to maintaining background resistance levels.

## Supplemental Material

(324 KB) PDF
