# Supplementary material for: Selective Pressure of Antibiotic Pollution on Bacteria of Importance to Public Health
Source: Environ Health Perspect. 2012 May 8;120(8):1100–6. doi: 10.1289/ehp.1104650 (PMC3440082; doi:10.1289/ehp.1104650)
Supplement: (324 KB) PDF [file ehp.1104650.s001.pdf]

SUPPLEMENTAL MATERIAL

Selective Pressure of Antibiotic Pollution on Bacteria of Importance to Public Health

Alfredo Tello[1*], Brian Austin[1], Trevor C. Telfer[1]

Institute of Aquaculture, University of Stirling, Stirlingshire, United Kingdom, FK9 4LA

*Corresponding author. Mailing Address: Institute of Aquaculture, University of Stirling, Stirlingshire, UK, FK9 4LA. E-mail address: alfredotello@gmail.com; Tel: +44(0)1786 467893; Fax: +44(0)1786 472133.

Table of Contents

Supplemental Material, Section 1 2
Correlation structure of MIC datasets 2
Supplemental Material, Figure S1 2

Supplemental Material, Table S1 3

Supplemental Material, Section 2 6
Evidence for growth of selected genera in the environment 6
Supplemental Material, Table S2 7

References 8

Supplemental Material, Section S1

Correlation structure of MIC datasets

An implicit assumption in the use of SSDs is that taxa used to derive them represent independent observations drawn from a random distribution (Suter et al. 2002). To minimize the lack of independence between observations, several methods and researchers recommend combining the endpoints from co-generic species (see Suter et al. 2002). Mantel tests (Mantel 1967; Legendre and Legendre 1998) were used to explore the correlation between evolutionary distances and pairwise differences in median MICs between taxa for each antibiotic dataset. Figure 1 shows Mantel correlograms for each antibiotic, along with the Mantel correlation coefficient for the entire dataset in the upper left hand side of each graph (i.e., $\mathbf{R_M}$). Grouping data from co – generic species to minimize the lack of independence before SSD derivation seemed advisable for the ciprofloxacin dataset, whereas there did not seem to be a statistical ground for doing so in the erythromycin and tetracycline datasets. In this study we decided to group co-generic species in all datasets in order to apply a consistent methodology in the derivation of SSDs for all three antibiotics that would facilitate the subsequent interpretation and discussion of results.

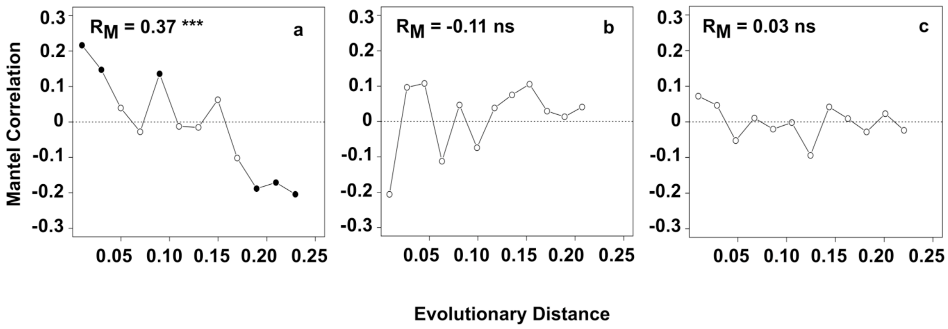

Supplemental Material, Figure S1. Mantel correlograms descrbing the correlation between MIC_{50} and evolutionary distance for ciprofloxacin (a), erythromycin (b) and tetracycline (c). Solid dots represent a significant correlation at the corresponding evolutionary distance ($\alpha = 0.05$). Significance tests are based on 5000 permutations.

Supplemental Material, Table S1

Taxa represented in EUCAST MIC datasets. Cross (+) and dash (-) symbols indicate presence or absence of each entry in the given EUCAST MIC antibiotic dataset, respectively. The All-species Living Tree Project names of the subset of species which were represented in the LTP 16S rRNA database are given in the 'LTP Name' column. MIC data from co – generic species in each dataset were pooled to derive SSDs.

Species	Ciprofloxacin	Erythromycin	Tetracycline	LTP Name
Acinetobacter anitratus	+	-	-	
Acinetobacter baumanii	+	-	+	AcnBau29
Acinetobacter lwoffii	+	-	-	AcnLwo34
Acinetobacter calcoaceticus	+	-	-	AcnCal77
Acinetobacter spp	+	-	+	
Alcaligenes xylosoxidans	+	-	-	
Bacteroides fragilis	+	-	-	BcdFra36
Bacteroides fragilis group	+	+	-	
Bacteroides ovatus	-	-	-	BcdOvat8
Bacteroides thetaiotaomicron	-	-	-	BcdThe32
Bacteroides vulgatus	-	-	-	BcdVulg9
Burkholderia cepacia	+	-	-	BurCe154
Bifidobacterium longum	-	+	+	BifLon13
Bifidobacterium pseudolongum	-	-	+	BifPseu8
Bifidobacterium thermophilum	-	-	+	
Campylobacter coli	+	+	+	CampCol5
Campylobacter jejuni	+	+	+	CamJejun
Chryseobacterium meningosepticum	+	-	-	
Chryseobacterium spp	+	-	-	
Citrobacter braakii	-	-	+	CitBraak
Citrobacter freundii	-	-	+	CitFre32
Citrobacter koseri	-	-	+	
Citrobacter spp	+	-	+	
Clostridium difficile	-	+	+	CloDiff5
Clostridium perfringens	-	+	-	CloPe172
Enterobacter aerogenes	+	-	+	EntAero9
Enterobacter agglomerans	+	-	+	
Enterobacter cloacae	+	-	+	EntClo58
Enterobacter dissolvens	+	-	+	EntClo52
Enterobacter spp	+	-	+	
Enterococcus avium	-	-	-	EnrAviu7
Enterococcus casseliflavus	-	-	-	EnrCas20
Enterococcus faecalis	+	+	+	EnrFa149
Enterococcus faecium	+	+	+	EnrFa232
Enterococcus gallinarum	-	-	-	EnrGall8

Supplemental Material, Table S1 (continued)

Species	Ciprofloxacin	Erythromycin	Tetracycline	LTP Name
Enterococcus hirae	-	+	+	EnrHir11
Escherichia coli	+	-	+	EschCo52
Haemophilus influenzae	+	+	+	HaeIn157
Haemophilus parainfluenzae	+	+	+	HaePara4
Hafnia alvei	+	-	+	HafAlv15
Helicobacter pylori	+	-	+	HelPyl55
Klebsiella oxytoca	+	-	+	KleOxy19
Klebsiella pneumoniae	+	-	+	KlePn101
Klebsiella spp	+	-	+	
Kluyvera spp	+	-	+	
Lactobacillus lactis	-	+	+	
Legionella pneumophila	+	+	-	LgnPne10
Listeria monocytogenes	+	-	+	LstMon13
Manhemia haemolytica	-	-	+	ManHaem8
Moraxella catarrhalis	+	+	+	MorCatar
Morganella morganii	+	-	+	MrgMor25
Mycobacterium tuberculosis	+	-	-	MycTube9
Neisseria gonorrhoeae	+	+	+	NeiGono5
Neisseria meningitidis	+	-	+	
Pasteurella multocida	+	+	+	PsuMult6
Peptostreptococcus spp	-	+	-	
Propionibacterium acnes	-	-	+	ProAcn42
Proteus mirabilis	+	-	+	PtsMir19
Proteus vulgaris	+	-	+	PtsVulg9
Proteus spp	-	-	+	
Providencia rettgeri	-	-	-	PrvRett3
Providencia stuartii	+	-	-	PrvStua5
Providencia spp	+	-	-	
Pseudomonas aureginosa	+	-	+	PseAe290
Pseudomonas fluorescens	+	-	-	PseFl192
Raoultella spp	+	-	+	
Raoultella spp+	-	-	+	
Salmonella enteritidis	-	-	-	
Salmonella paratyphi	-	-	-	
Salmonella typhi	-	-	-	
Salmonella typhymurium	-	-	-	
Salmonella spp	+	-	+	
Serratia liquefaciens	+	-	+	SerLiqu3
Serratia marcescens	+	-	+	SerMa116
Serratia spp	+	-	+	
Shigella sonnei	-	-	-	StaAur61
Staphylococcus aureus	+	+	+	
Staphylococcus aureus MRSA	+	+	+	
Staphylococcus aureus MSSA	+	+	+	
Staphylococcus auricularis	+	-	-	StaAuri2
Staphylococcus capitis	+	+	-	StaCap14

Supplemental Material, Table S1 (continued)

Species	Ciprofloxacin	Erythromycin	Tetracycline	LTP Name
Staphylococcus coagulase negative	+	+	+	
Staphylococcus coagulase negative MRSE	+	+	+	
Staphylococcus cohnii	+	-	-	StaCohn9
Staphylococcus epidermidis	+	+	+	StaEpi72
Staphylococcus epidermidis MSSE	+	+	+	
Staphylococcus haemolyticus	+	+	+	StaHae18
Staphylococcus hominis	+	+	-	StaHom26
Staphylococcus hyicus	+	+	-	StaHyicu
Staphylococcus intermedius	+	+	-	StaInte4
Staphylococcus lugdunensis	+	-	-	StaLugd4
Staphylococcus saprophyticus	+	+	+	StaSap31
Staphylococcus sciuri	+	-	-	StaSci15
Staphylococcus simulans	+	-	-	StaSimul
Staphylococcus warneri	+	+	-	StaWarne
Staphylococcus xylosis	+	-	-	StaXyl10
Strenotrophomonas maltophila	+	-	+	SteMa216
Streptococcus acidominimus	+	-	-	
Streptococcus agalactiae	+	+	+	StpAga25
Staphylococcus anginosus	+	+	+	StpAng35
Streptococcus bovis	+	-	-	
Streptococcus constellatus	+	-	-	StpCon17
Streptococcus equinus	+	-	-	StpEqui4
Streptococcus gordonii	+	-	-	StpGor11
Streptococcus group C	+	-	-	
Streptococcus group G	+	+	+	
Streptococcus intermedius	+	-	-	StpInte8
Streptococcus milleri	+	+	-	
Streptococcus mitis	+	-	-	StpMit32
Streptococcus oralis	+	+	+	StpOra18
Streptococcus parasanguis	+	-	-	StpPar15
Streptococcus pneumoniae	+	+	+	StpPne46
Streptococcus pyogenes	+	+	+	StpPyo39
Streptococcus salivarius	+	-	-	StpSali6
Streptococcus sanguinis	+	-	-	StpSan11
Streptococcus thermophilus	-	+	+	
Streptococcus uberis	+	-	-	StpUber5
Streptococcus viridans	+	+	+	
Yersinia spp	+	-	+	
**Total**	**91**	**40**	**67**	**79**
**16S rRNA sequences**	**63 (70%)**	**28 (70%)**	**43 (64%)**	

Supplemental Material, Section S2

Evidence for growth of selected genera in the environment

There is evidence to suggest that several bacteria of importance to public health may, under certain conditions, grow in different environments. Hendricks (1972), for eample, showed that some *Enterobacteriaceae* could grow in water collected downstream of a municipal sewage facility at temperatures as low as 5°C. Gibbs et al. (1997) reported years later the re-growth of faecal coliforms and *Salmonella* in biosolids and soil ammended with biosolids. The populations of *E. coli* in manured soils can be very dynamic (Topp et al. 2003) and Inglis et al. (2010) recently showed that *Campylobacter* can persist for long periods of time in compost, which may suggest cryptic growth. Enterococci have been shown to grow in municipal oxidation ponds (Moriarty et al. 2008), and the facultative intracellular pathogen *Listeria monocytogenes* has been shown to be widespread in certain catchments (Lyautey et al. 2007) and able to grow in soil suspensions characteristic of certain organic fractions (Sidorenko et al. 2006). Staphylococci, including *Staphylococcus aureus*, have been isolated from marine water samples (Gunn and Colwell 1983), and methicillin – resistant *Staphylococcus aureus* was recently isolated from marine water and intertidal sand from beaches on the west coast of the USA (Soge et al. 2009). *S. aureus* has also been shown to be capable of growth in sterile soil (Liang et al. 1982), suggesting that it might be possible for it to grow in this environment under conditions of low competition. Ayyadurai et al. (2008) recently demonstrated that *Yersinia pestis* remained viable and fully virulent in humid sand for 40 weeks. *Clostridium difficile* is widely distributed in the environment (Al Saif and Brazier 1996), and it is not unreasonable to speculate that there may be niches that could

support its sporulation. Collectively, these studies highlight there is a potential for growth in bacteria of clinical relevance in different environments and under varying biological and physicochemical conditions, even if only during a short temporal window. In the presence of antibiotics, wild-type populations may be inhibited to various extents, increasing the prevalence of resistance. Full references upon which we based our decision to include genera for SSD derivation are given in Table S2.

Supplemental Material, Table S2

Bacterial genera represented in the SSDs. References provide either direct evidence of growth in the environment or evidence that suggests that under certain conditions it is possible for growth to occur.

	Genus	Reference
1	*Acinetobacter*	Madigan et al. 2009
2	*Alcaligenes*	Agerso et al. 2005; Madigan et al. 2009
3	*Burkholderia*	Madigan et al. 2009
4	*Campylobacter*	Brandl et al. 2004; Inglis et al. 2010
5	*Chryseobacterium*	Vandamme et al. 1994
6	*Citrobacter*	Madigan et al. 2009; Kitts et al. 1994
7	*Clostridium*	Al Saif and Brazier 1996; Madigan et al. 2009
8	*Enterobacter*	Hendricks 1972; Rattray et al. 1995; Hernandez et al. 1998
9	*Enterococcus*	Mundt 1961/1963; Desmarais 2002; Moriarty et al. 2008
10	*Escherichia*	Hendricks 1972; Gibbs et al. 1997; Topp et al. 2003; Zaleski et al. 2005
11	*Hafnia*	Jand 2006
12	*Klebsiella*	Liang et al. 1982; Zadoks et al. 2011 (and references therein)
13	*Kluyvera*	Janda 2006
14	*Legionella*	Fliermans et al. 1981
15	*Listeria*	Sidorenko et al. 2006; Lyautey et al. 2007
16	*Morganella*	Kitts et al. 1994
17	*Pasteurella*	Bredy and Botzler 1989
18	*Proteus*	Hendricks 1972
19	*Providencia*	Kitts et al. 1994
20	*Pseudomonas*	Madigan et al. 2009
21	*Raoultella*	Zadoks et al. 2011 (and references therein)
22	*Salmonella*	Liang et al. 1982; Gibbs et al. 1997
23	*Serratia*	Madigan et al. 2009
24	*Staphylococcus*	Liang et al. 1982; Gunn and Colwell 1983; Soge et al. 2009
25	*Stenotrophomonas*	Bollet et al. 1995
26	*Streptococcus*	Gledhill and Casida 1969
27	*Yersinia*	Sidorenko et al. 2006; Ayyadurai et al. 2008

References

Agersø Y, Sandvang D. 2005. Class 1 Integrons and Tetracycline Resistance Genes in Alcaligenes, Arthrobacter, and Pseudomonas spp. Isolated from Pigsties and Manured Soil. Appl Environ Microbiol71:7941-7947.

Al Saif N, Brazier JS. 1996. The distribution of Clostridium difficile in the environment of South Wales. J Med Microbiol 45:133-137.

Ayyadurai S, Houhamdi L, Lepidi H, Nappez C, Raoult D, Drancourt M. 2008.Long-term persistence of virulent Yersinia pestis in soil. Microbiology 154:2865-2871.

Bollet C, Davin-Regli A, De Micco A. 1995. A Simple Method for Selective Isolation of Stenotrophomonas maltophilia from Environmental Samples. Appl Environ Microbiol 61:1653-1654.

Brandl MT, Haxo AF, Bates AH, Mandrell RE. 2004. Comparison of Survival of Campylobacter jejuni in the Phyllosphere with That in the Rhizosphere of Spinach and Radish Plants. Appl Environ Microbiol 70:1182-1189.

Bredy JP, Botzler RG. 1989. The effects of six environmental variables on Pasteurella multocida populations in water. J Wildl Dis 25:232-239.

Desmarais TR, Solo-Gabriele HM, Palmer CJ. 2002. Influence of soil on fecal indicator organisms in a tidally influenced subtropical environment. Appl Environ Microbiol 68:1165-1172.

Fliermans CB, Cherry WB, Orrison LH, Smith SJ, Tison DL, Pope, DH. 1981. Ecological distribution of Legionella pneumophila. Appl Environ Microbiol 41:9-16.

Gibbs RA, Hu CJ, Ho GE, Unkovick I. 1997. Regrowth of fecal coliforms and salmonella in stored biosolids and soil amended with biosolids. Water Sci Technol 35:269-275.

Gledhill WE, Casida LE. 1969. Predominant Catalase-negative Soil Bacteria. I. Streptococcal Population Indigenous to Soil1. Appl Microbiol 17:208-213.

Gunn BA, Colwell RR. 1983. Numerical Taxonomy of Staphylococci Isolated from the Marine Environment. Int J Syst Bacteriol 33:751-759.

Hendricks CW. 1972. Enteric Bacterial Growth Rates in River Water. Appl Microbiol 24:168-174.

Hernandez A, Mellado RP, Martínez JL. 1998. Metal accumulation and vanadium-induced multidrug resistance by environmental isolates of Escherichia hermannii and Enterobacter cloacae. Appl Environ Microbiol 64:4317-4320

Inglis GD, McAllister TA, Larney FJ, Topp E. 2010. Prolonged Survival of Campylobacter Species in Bovine Manure Compost. Appl Environ Microbiol 76:1110-1119.

Janda JM, Abbott SL. 2006. The Genus Hafnia: from Soup to Nuts. Clin Microbiol Rev 19:12-18.

Janda JM. 2006. New Members of the Family Enterobacteriaceae. In M. Dworkin & S. Falkow, eds. The prokaryotes: a handbook on the biology of bacteria. New York: Springer. Pp. 5 - 40.

Kitts CL, Cunningham, DP, Unkefer PJ. 1994. Isolation of three hexahydro-1,3,5-trinitro-1,3,5-triazine-degrading species of the family Enterobacteriaceae from nitramine explosive-contaminated soil. Appl Environ Microbiol 60:4608-4611.

Legender P, Legendre L. 1998. Numerical Ecology. Amsterdam:Elsevier Science B.V., 1-853.

Liang LN, Sinclair JL, Mallory LM, Alexander M. 1982. Fate in model ecosystems of microbial species of potential use in genetic engineering. Appl Environ Microbiol 44:708-714.

Lyautey E, Jackson CR, Cayrou J, Rols, JL, Garabétian F. 2005. Distribution and characteristics of Listeria monocytogenes isolates from surface waters of the South Nation River watershed, Ontario, Canada. Appl Environ Microbiol 73:5401-5410.

Madigan MT, Martinko JM, Dunlap PV, Clark DP. 2009. Brock Biology of Microorganisms. San Francisco:Pearson Benjamin Cummings, 1-1061.

Mantel N. 1967. The detection of disease clustering and a generalized regression approach. Cancer Res 27:209-220.

Moriarty E, Nourozi F, Robson B, Wood D, Gilpin B. 2008. Evidence for growth on enterococci in municipal oxidation ponds obtained using antibiotic resistance analysis. Appl Environ Microbiol 74:7204-7210.

Mundt JO. 1963. Occurrence of Enterococci on Plants in a Wild Environment. Appl Microbiol 11:141-144.

Mundt JO. 1961. Occurrence of Enterococci: Bud, Blossom, and Soil Studies. Appl Microbiol 9:541-544.

Rattray EA, Prosser JI, Glover LA, Killham K. 1995. Characterization of rhizosphere colonization by luminescent Enterobacter cloacae at the population and single-cell levels. Appl Environ Microbiol 61:2950-2957.

Sidorenko ML, Buzoleva LS, Kostenkov NM. 2006. The effect of soil properties on the preservation and reproduction of Listeria and Yersinia. Eurasian Soil Sci 39:211-217.

Suter II GW, Traas TP, Posthuma L. 2002. Issues and practices in the derivation of species sensitivity distributions. In: Species sensitivity distributions in ecotoxicology (Posthuma L, Suter II GW, Traas TP, eds). Florida:Lewis Publishers, 437-473.

Topp E, Welsh M, Tien YC, Dang A, Lazarovits G, Conn K, et al. 2003. Strain-dependent variability in growth and survival of Escherichia coli in agricultural soil. FEMS Microbiol Lett 44 44:303-8.

Vandamme P, Bernardet JF, Segers P, Kersters K, Holmes B. 1994. New perspectives in the classification of the flavobacteria: Description of Chryseobacterium gen. nov., Bergeyella gen. nov., and Empedobacter nom. rev. Int J Syst Bacteriol 44:827-831.

Zadoks RN, Griffiths HM, Munoz MA, Ahlstrom C, Bennett GJ, Thomas E, et al. 2011. Sources of Klebsiella and Raoultella species on dairy farms: be careful where you walk. J Dairy Sci 94:1045-1051.

Zaleski KJ, Josephson KL, Gerba CP, Pepper IL. 2005. Potential Regrowth and Recolonization of Salmonellae and Indicators in Biosolids and Biosolid-Amended Soil. Appl Environ Microbiol 71:3701-3708.
